# Cost-effectiveness of Implementing Smoking Cessation Interventions for Patients With Cancer

**DOI:** 10.1001/jamanetworkopen.2022.16362

**Published:** 2022-06-09

**Authors:** Douglas E. Levy, Susan Regan, Giselle K. Perez, Alona Muzikansky, Emily R. Friedman, Julia Rabin, Nancy A. Rigotti, Jamie S. Ostroff, Elyse R. Park

**Affiliations:** 1 Mongan Institute Health Policy Research Center, Massachusetts General Hospital, Boston; 2 Tobacco Research and Treatment Center, Massachusetts General Hospital, Boston; 3 Harvard Medical School, Boston, Massachusetts; 4 Division of General Internal Medicine, Massachusetts General Hospital, Boston; 5 Health Promotion and Resiliency Intervention Research Program, Massachusetts General Hospital, Boston; 6 Department of Psychiatry, Massachusetts General Hospital, Boston; 7 MGH Biostatistics Center, Massachusetts General Hospital, Boston; 8 Department of Psychology, University of Cincinnati, Cincinnati, Ohio; 9 Department of Psychiatry & Behavioral Sciences, Memorial Sloan Kettering Cancer Center, New York, New York

## Abstract

**Importance:**

Guidelines recommend cancer care clinicians offer smoking cessation treatment. Cost analyses will help stakeholders understand and plan for implementation of cessation programs.

**Objective:**

To estimate the incremental cost per quit (ICQ) of adopting an intensive smoking cessation intervention among patients undergoing treatment at cancer care clinics, from a clinic perspective.

**Design, Setting, and Participants:**

This economic evaluation, a secondary analysis of the Smokefree Support Study (conducted 2013-2018; completed 2021), used microcosting methods and sensitivity analyses to estimate the ICQ of the interventions. Participants included patients undergoing treatment for a broad range of solid tumors and lymphomas who reported current smoking and were receiving care at cancer care clinics within 2 academic medical centers.

**Exposures:**

Intensive smoking cessation treatment (up to 11 counseling sessions with free medications), standard of care (up to 4 counseling sessions with medication advice), or usual care (referral to the state quitline).

**Main Outcomes and Measures:**

Total costs, component-specific costs, and the ICQ of the intensive smoking cessation treatment relative to both standard of care (comparator in the parent randomized trial) and usual care (a common comparator outside this trial) were calculated. Overall and post hoc site-specific estimates are provided. Because usual care was not included in the parent trial, sensitivity analyses were conducted to assess how assumptions about usual care quit rates affected study outcomes (ie, base case [from a published smoking cessation trial among patients with thoracic cancer], best case, and conservative case scenarios).

**Results:**

The per-patient costs of offering intensive smoking cessation treatment, standard of care, and usual care were $1989, $1482, and $0, respectively. For intensive treatment, the dominant costs were treatment (35%), staff supervision (26%), and patient enrollment (24%). Relative to standard of care, intensive treatment had an overall ICQ of $3906, and one site had an ICQ of $2892. Relative to usual care, intensive treatment had an ICQ of $9866 overall (base case), although at one site, the ICQ was $5408 (base case) and $3786 (best case).

**Conclusions and Relevance:**

In this economic evaluation study, implementation of an intensive smoking cessation treatment intervention was moderately to highly cost-effective, depending on existing smoking cessation services in place.

## Key Points

**Question:**

What is the incremental cost per quit of offering intensive smoking cessation treatment in cancer care clinics relative to standard of care and usual care?

**Findings:**

In this economic evaluation, the intensive smoking cessation treatment program had an incremental cost per quit of $3906 relative to standard of care and $9866 relative to usual care. Site-specific analyses suggest that the intensive treatment program may achieve incremental costs per quit as low as $2892 and $5408 vs standard and usual care, respectively.

**Meaning:**

In this study, the incremental cost per quit of an intensive smoking cessation treatment program was comparable with other cessation programs and should be considered as a tobacco treatment model in cancer care.

## Teaser

This economic evaluation estimates the incremental cost per quit of adopting an intensive smoking cessation intervention among patients undergoing treatment at cancer care clinics, from a clinic perspective.

## Introduction

Leading scientific, academic, professional, and advocacy organizations, including the National Cancer Institute (NCI), the American Association of Cancer Researchers, the American Society for Clinical Oncology, the National Comprehensive Cancer Center Network (NCCN), and the American Cancer Society, strongly recommend provision of smoking cessation treatment to all those diagnosed with cancer who smoke.^1,2,3,4,5^ Cigarette smoking is associated with poorer clinical outcomes for patients with cancer, including increased risk of second primary cancers, disease recurrence, treatment-related toxic effects, and mortality.^6^ Despite the well-established risks and professional consensus, tobacco cessation treatment is not provided consistently to patients with cancer.^7,8^ Uncertainty about operation costs may be a barrier for stakeholders contemplating the addition of tobacco cessation treatment initiatives.

Over the course of cancer care, there are many opportunities to support smoking cessation. The Smokefree Support Study, a randomized comparative effectiveness trial, identified a promising approach for smoking cessation treatment delivery in the cancer care setting. This trial compared 2 approaches to supporting smoking cessation among patients initiating cancer care: an intensive smoking cessation treatment program (up to 11 counseling sessions and free cessation medication) vs standard of care (up to 4 counseling sessions, including medication advice, based on the NCCN Smoking Cessation Guideline Recommendation).^9^ The study found the intensive treatment outperformed standard of care with cotinine confirmed 7-day point prevalence abstinence at 6 months of 34.5% and 21.5%, respectively (difference, 13.0% [95% CI, 3.0%-23.3%]).^10^

The trial included a prespecified cost-effectiveness analysis with cost data collected alongside the other outcomes with the goal of informing future stakeholders about the resource demands of implementing the intervention. In this study, we present that analysis, assessing the incremental cost per quit (ICQ) of intensive smoking cessation treatment vs standard treatment (measured in the trial) and usual care (quitline referral, estimated from another trial) from a cancer care center’s perspective.

## Methods

### Smokefree Support Study Overview

Conducted from November 2013 to January 2018, the Smokefree Support Study was a 2-site randomized clinical comparative effectiveness trial testing the offer of a smoking cessation treatment program providing counseling and Food and Drug Administration–approved smoking cessation medications to patients undergoing treatment for a recently diagnosed breast, gastrointestinal, genitourinary, gynecological, head and neck, lymphoma, lung, or melanoma cancer. The study protocol and main results have been published elsewhere (NCT01871506).^9,10^ Briefly, a convenience sample of patients initiating cancer care at 2 academic cancer centers were screened for tobacco use, and patients who reported current smoking were invited to participate in the study. After consent and enrollment, patients were randomized to offer of either standard of care (ST, ie, up to 4 weekly brief telephone counseling sessions plus advice on the use of smoking cessation medications) or intensive smoking cessation treatment (IT, ie, up to 11 brief telephone counseling sessions and provision of up to 12 weeks of smoking cessation medications). Patients in the IT group were provided options for receipt of cessation medications, including varenicline, bupropion, or nicotine replacement therapy (patch and/or gum or lozenges), as agreed by smoking cessation counselor and patient. Smoking cessation was defined as biochemically confirmed 7-day point prevalence abstinence at 6-month follow-up, the standard outcome for smoking cessation trials.^11^ The research was approved by the institutional review boards of the participating sites, and written informed consent was obtained from all study participants. This economic evaluation followed the Consolidated Health Economic Evaluation Reporting Standards (CHEERS) reporting guideline.^13^

### Cost Analysis Overview

The cost-effectiveness analysis estimated the ICQ over the course of treatment and follow-up (6 months) of IT vs ST. In addition, the analysis estimated the ICQ of IT vs usual care (referral to the state tobacco quitline), a more generalizable comparator. There was no usual care arm in the trial, so quit rates for the usual care scenario were derived by sensitivity analyses. Base case usual care quit rates were drawn from a prior smoking cessation study among patients in thoracic surgery and oncology conducted in 2008 to 2009.^12^ That study’s inclusion and exclusion criteria were similar to the Smokefree Support Study, with the exception of the narrower eligibility diagnosis. In the prior study, biochemically verified 7-day point prevalence abstinence at 12-week follow-up among patients receiving usual care was 14.3%.

A microcosting approach was used to assess costs across 4 broad categories: training costs for intervention personnel, patient identification and enrollment, treatment costs (counseling, medication, documentation), and costs for other resources, such as printed materials and office space. Costs were measured separately for the 2 sites because they had different workflows and staffing; both overall and deidentified site-specific costs were assessed. All costs are reported in 2018 US dollars.

### Cost Components

#### Training and Supervision

Counselors received tobacco treatment training (eTable 1 in the Supplement). For general tobacco treatment training provided outside the cancer center (ie, basic skills for working with smokers, tobacco treatment specialist training), costs included course fees and the counselors’ time. For trial-specific training within the cancer center (ie, counseling protocol, motivational interview training, ongoing supervision), costs included the time spent by trainers, who were psychologists, as well as by counselors. With the exception of ongoing supervision, it was assumed that these were one-time costs. Economic evaluations taking a long-run perspective would exclude these one-time costs; however, a goal of this analysis is to prepare decision-makers for the cost of launching the programs under study, so they are included.

#### Patient Identification and Enrollment

The time staff dedicated to identifying and enrolling patients in the intervention was tracked (eTable 2 in the Supplement).^9^ It was assumed that the effort required to identify smokers eligible for this smoking cessation trial and explain the program would roughly approximate the time it would take to identify and enroll patients in an analogous nonresearch clinical program. Enrollment time excluded research-specific tasks such as obtaining informed consent and data collection for research outcomes.

#### Treatment

##### Counseling Delivery

Counselors’ time included scheduling, call attempts and voicemails, and the delivery of intervention sessions. Intervention delivery included time preparing for, delivering, and documenting counseling sessions. Many patients did not complete all assigned counseling sessions; only the cost of completed sessions was included (eTable 3 in the Supplement).

##### Medication

Medication included the costs of the medications, delivery, and clinician time for both prescribing and medication reconciliation (eTable 4 in the Supplement). The cost of prescription medications was estimated using average wholesale price for 2018 minus 20%,^14^ as recommended for single source medications by the ISPOR Drug Cost Task Force.^15^ The cost of nicotine replacement therapy was based on the lowest online price available during the time the study was under way.

#### Other Costs

Other costs included the cost of printed materials used in enrolling patients and the cost of office space for staff (eTable 5 in the Supplement). The cost of printed materials was calculated using study records. The cost of office space was based on study institutions’ nonclinical office space rate.

#### Usual Care

Under usual care, it was assumed that medical assistants would send referrals for 32% of patients to the state tobacco quitline, and referrals would take approximately 2 minutes.^16,17^ Under these assumptions, the cost of usual care is less than $1 per patient and was deemed ignorable.

#### Wages

Personnel included psychologists who provided supervision; nurse practitioners who conducted enrollment, provided counseling, and prescribed medication (Site A); social workers who provided counseling (Site B); and research assistants who conducted enrollment and managed medication shipments (both sites). In a clinical program, medical assistants might take on the research assistants’ role. Costs of personnel time were estimated by multiplying the frequency and duration of study tasks by the appropriate personnel’s wages using national median wages in 2018 obtained from the US Bureau of Labor Statistics and included fringe benefits (eTable 6 in the Supplement).^18^ The one exception was psychologists’ wages, where the national 90th percentile value was used to better reflect the specialization and advanced expertise of the psychologists employed in the trial.

### Statistical Analysis

#### ICQ

ICQ was estimated as the cost per patient receiving IT minus the cost per patient receiving the comparator treatment, divided by the quit rate for the IT group minus the quit rate for the comparator group. The comparator group was either ST or usual care. Quit rates for IT and ST were drawn from biochemically confirmed 7-day point prevalence abstinence measured at 6 months in the trial.

#### Probabilistic Sensitivity Analyses

To generate confidence bounds on the incremental cost estimates, Monte Carlo methods were used. The incremental cost was calculated in each of 100 000 simulations. For each simulation, the parameters for calculating incremental cost were drawn from appropriate probability distributions reflecting the uncertainty in those parameters (eTable 7 in the Supplement). There were 2 forms of uncertainty accounted for in the models, depending on the parameter. Some costs, eg, wages, do not vary stochastically but may be higher or lower depending on the study setting. In these cases, it was assumed that costs would be within 20% of the base case value, with the percentage difference varying according to a β distribution. Other costs were estimated from study data, and the distribution of those values was based on observed variability. Monte Carlo simulations were performed with Stata version 15.1 (StataCorp).

#### Usual Care Sensitivity Analyses

Usual care smoking cessation rates were obtained from a prior smoking cessation trial among thoracic surgery and oncology patients.^12^ Relative to the Smokefree Support Study, this cessation rate is likely conservative (ie, high and therefore less favorable to IT) because more relapse would be expected at 6-month follow-up (Smokefree Support Study) than at 12-week follow-up and because patients with lung cancer are typically more motivated to quit than patients with other cancers.^10^ Nevertheless, a maximally conservative scenario setting the usual care quit rate equal to the quit rate for ST (21.5%) and a best-case scenario (most favorable to IT) assuming a quit rate equal to general adult population quit rate (4.2%)^19^ were also included.

#### ICQ Confidence Intervals

Where appropriate, the Fieller method was used to approximate confidence intervals of ICQ estimates.^20,21,22^ Point estimates and confidence intervals based on Fieller method were calculated using Excel version 16.60 (Microsoft Corp).

## Results

### Intervention Costs

Over the 4-year study period, the IT intervention had a mean cost of $1989 per patient (Table). The intervention itself (ie, counseling and medications) was the largest component of the total cost, accounting for 35% of per-patient spending, with 19% for counseling delivery and 16% for provision of medications. Identification and enrollment of patients accounted for 24% of costs, while ongoing counselor supervision made up 26% of costs. The remaining costs consisted of initial training (5%) and other resources (10%).

**Table.** Costs and Incremental Costs per Quit

Component	Site-specific costs per patient by type of smoking cessation program (% total per patient cost), $^a^							Incremental value of each comparison, $^b^						
	IT			ST			UC	IT vs ST				IT vs UC		
	Overall	Site A	Site B	Overall	Site A	Site B	Overall	Overall		Site A	Site B	Overall	Site A	Site B
Training														
Initial	99 (5)	165 (5)	60 (5)	99 (7)	165 (6)	60 (8)	0	0	0		0	99	165	60
Ongoing supervision	511 (26)	818 (25)	332 (26)	511 (34)	818 (31)	332 (42)	0	0	0		0	511	818	332
Patient identification and enrollment	479 (24)	1039 (32)	153 (12)	479 (32)	1039 (39)	153 (19)	0	0	0		0	479	1039	153
Treatment														
Counseling delivery	369 (19)	736 (23)	155 (12)	189 (13)	392 (15)	72 (9)	0	183	344		83	369	736	155
Medications	327 (16)	215 (7)	392 (31)	0	0	0	0	325	215		392	327	215	392
Other resources	205 (10)	242 (8)	183 (14)	205 (14)	242 (9)	183 (23)	0	0	0		0	205	242	183
Total cost per patient, $	1989	3216	1276	1482	2656	800	0	508	559		475	1989	3216	1276
Quit rate, %^c^	34.5	28.3	37.9	21.5	21.6	21.4	14.3	13.0	6.7		16.5	20.2	14.0	23.6
Incremental cost per quit (ICQ)	NA	NA	NA	NA	NA	NA	NA	3906	8316		2892	9866	22 969	5408

Abbreviations: IT, intensive treatment; NA, not applicable; ST, standard of care; UC, usual care.

^a^ Totals and percentages may not add up perfectly due to rounding.

^b^ Incremental values are the value of IT minus the value of its comparator (ST or UC).

^c^ Quit rates for IT and ST are drawn from the Smokefree Support Study. The quit rate for UC uses the base case quit rate drawn from Park et al.^13^

Over the same period, the mean cost in the ST group was $1482 per patient. Differences with IT were driven solely by the smaller number of counseling sessions delivered and the fact that no medications were provided under ST. Although up to 4 counseling sessions were offered under ST compared with up to 11 offered under IT, average ST counseling costs were more than half the IT counseling costs because attendance at counseling sessions waned over time and the duration of later sessions was shorter (eTable 3 in the Supplement).

Notably, costs differed by site because of patient volume and preferences, staffing, and clinical workflows (eTables 2-6 in the Supplement). Site A costs for training and counseling were higher because the counselors were nurse practitioners compared with social workers at Site B, and Site A had 3 counselors compared with Site B, which had 2, resulting in higher training costs. Enrollment costs were also higher at Site A. Site A had higher patient volume and screened more than twice as many patients as Site B. Of the total effort devoted to delivering counseling, the sites were similar in the time they devoted to patient contact, but Site A had additional institutional requirements for routine clinical documentation (results not shown). Medication costs per patient were higher at Site B because more patients received medications.

### ICQ of IT vs ST

**Figure 1.**
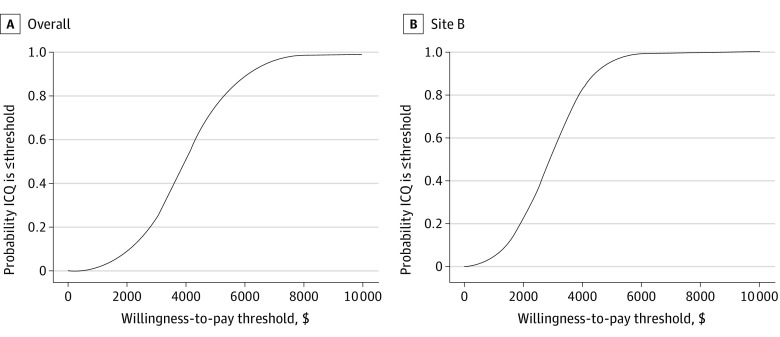
Likelihood of Cost-effectiveness of Intensive Treatment Relative to Standard-of-Care Treatment Across Willingness-to-Pay Thresholds ICQ indicates incremental cost per quit.

The overall ICQ of IT vs ST was $3906 (95% CI, $776-$7019) (Table). Site-specific ICQs were $8316 and $2892 for Sites A and B, respectively. Figure 1 presents the probability, based on estimated confidence intervals, that the ICQ was less than a range of willingness-to-pay thresholds that might be held by health care systems. Because the 95% CI for incremental effectiveness of IT at Site A substantially overlapped with 0, the Fieller method for calculating confidence intervals was not appropriate, and we omit Site A from Figure 1. There was near certainty that the ICQ was $10 000 or less, both overall and at Site B, and there was a 75.4% (overall) and 96.3% (Site B) chance that the ICQ was $5000 or less.

### ICQ of IT vs Usual Care

**Figure 2.**
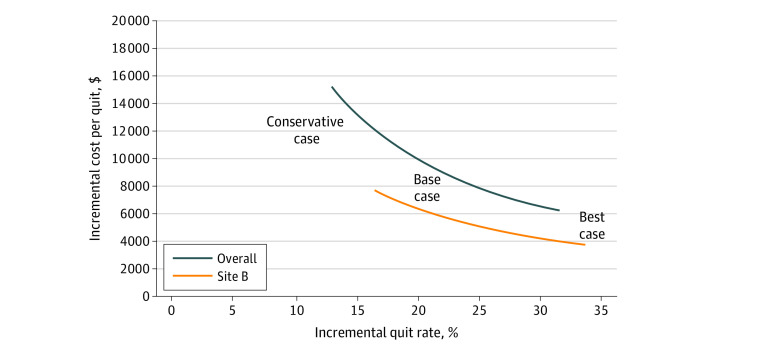
Incremental Cost per Quit of Intensive Treatment vs Usual Care as a Function of the Incremental Quit Rate of Intensive Treatment vs Usual Care Intensive treatment group quit rate was 34.5%; usual care quit rates were 21.5% in the conservative case; 14.3% in the base case, and 4.2% in the best case.

**Figure 3.**
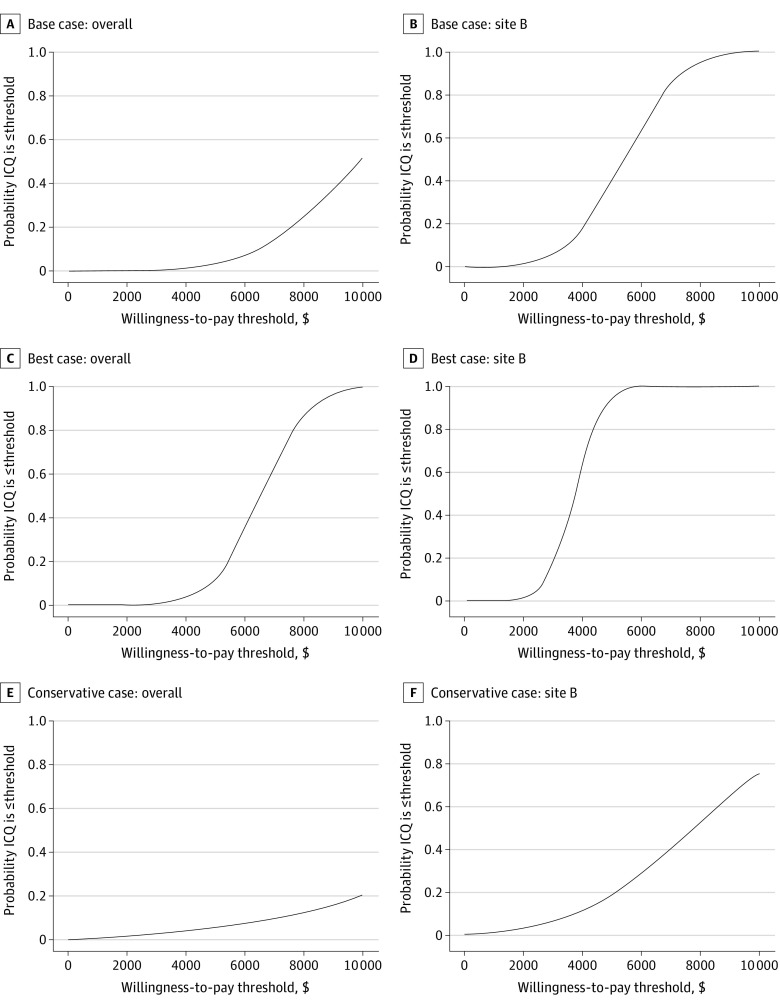
Likelihood of Cost-effectiveness of Intensive Treatment Relative to Comparator Across Willingness-to-Pay Thresholds by Assumed Usual Care Quit Rates ICQ indicates incremental cost per quit.

Under the base case, the incremental cost of IT relative to usual care was significantly higher than the incremental cost of IT relative to ST, but the incremental effectiveness under the base case was also higher (Table). Because the true quit rate for usual care is unknown, Figure 2 illustrates how the overall ICQ of IT vs usual care varies with incremental effectiveness, holding the incremental cost constant at $1989 (overall) and $1276 (Site B). Under the base case, the ICQ was $9866 overall and $5408 at Site B. In the best case scenario, where the usual care quit rate is equal to the overall population quit rate, the ICQ was $6314 overall and $3786 at Site B. In the most conservative case, where the usual care quit rate equals that of the ST arm, the ICQs were $15 300 overall and $7733 at Site B. Figure 3 illustrates the probabilities, based on estimated confidence intervals for each case, that the ICQs were less than a range of health systems’ willingness-to-pay thresholds. Under the base case, there was a 52.0% (overall) and 99.8% (Site B) chance that the ICQ was $10 000 or less and a 3.4% (overall) and 39.7% (Site B) chance that the ICQ was $5000 or less. Under the best case, there was a 12% (overall) or 94% (Site B) chance that the ICQ was $5000 or less. Under the conservative case, there was a 5.3% (overall) or 18.5% (Site B) chance that the ICQ was $5000 or less.

## Discussion

As assessed according to a preplanned cost-effectiveness analysis of the Smokefree Support Study, expanding the offer of standard of care smoking cessation treatment (up to 4 counseling sessions and medication advice) to the offer of intensive smoking cessation treatment (up to 11 counseling sessions plus provision of cessation medication) can be achieved at an expected ICQ of $3906. ST was chosen as the trial comparator to mirror the standard of care at Site A, NCCN guideline–recommended offer of at least 4 counseling sessions.^23^ However, this quality of care recommendation is not adopted as usual care at many institutions. When IT was compared with a base case usual care scenario anchored with data from a prior smoking cessation trial among patients in thoracic surgery and oncology, both the incremental cost and incremental quit rate were higher, but the higher relative increase in incremental cost resulted in an ICQ of $9866. The results from the prior study may not be a precisely analogous comparator. At worst, if quit rates for usual care were much higher (ie, the same as ST), the ICQ would be more than 50% higher. On the other hand, it is likely that the smoking prevalence and quit rates were higher in the prior study than they would be in a routine oncology care setting. For this reason and reasons previously noted, the resulting base case cost-effectiveness estimate is likely conservative. In a best case analysis, where the usual care group had a quit rate similar to the general population, the ICQ was $6314.

In comparison, other health system–based smoking cessation initiatives have ICQs relative to usual care ranging from $554 to $6267 in 2018 US dollars,^24,25,26,27,28,29,30^ although only 1 of these^24^ took place in the oncology setting. Four of these studies defined cessation using the same metric as the current study: abstinence at 6 months,^25,27,28,30^ which is the standard for smoking cessation trials. One used abstinence at 12 months.^29^ Prior research suggests there is relatively little relapse among those abstinent at 6 months follow-up.^31^ Two studies^24,26^ defined cessation as abstinence at 3 months (ICQ of $3353 and $531), which would be less conservative from a cost-effectiveness perspective. If the incremental quit rate were lower for these studies at 6 months than at 3 months, their ICQs would be higher.

The value of the IT intervention should be placed in the context of the clinical benefits conferred by smoking cessation in this patient population, benefits that were not measured in the study. Because cancer diagnoses and treatment courses in the study population were heterogeneous, we did not have adequate power to estimate costs or quit rates for patients with specific cancer diagnoses, and it was not feasible to estimate the incremental cost per life-year saved of our intervention. However, smoking cessation is known to benefit patients with cancer, improving cancer treatment outcomes and quality of life for patients with varied tumor types and stages. The 2020 Surgeon General’s report concluded that continued smoking among patients with cancer leads to adverse health outcomes, increased all-cause and cancer-specific mortality, and increased risk for second primary cancers known to be caused by cigarette smoking.^6^ Continuing tobacco use by patients receiving treatment for cancer can contribute to treatment failure, with costs estimated to exceed $10 000 per smoker.^32^ With this value as a benchmark, point estimates for the ICQs suggest initiating the IT intervention where ST had been in place would be cost saving. Initiating the IT intervention where usual care was previously in place could be cost saving, depending on existing staffing and workflows. It is nevertheless important to note these savings may accrue to payers rather than the cancer care center that bears the costs of IT; contracts between payers and clinician groups might need to be renegotiated so payers adequately compensate clinicians for the provision of smoking cessation treatment, thereby aligning each stakeholder’s incentives and ensuring centers do not forego offering smoking cessation treatment due to cost.

New health informatics technology may mitigate the costs of IT and improve cost-effectiveness in future implementations. Identifying and enrolling patients who reported current smoking incurred significant costs. All the enrollment costs included in the current study were personnel costs, although these efforts were partially facilitated by the institutions’ existing electronic health records systems (previously sunk costs) and clinical workflows. Most cancer centers have now adopted information technology systems that have the capability of automating the identification of patients and referral to smoking cessation services.^33^ After the cost of initial investment in such infrastructure along with frontline staff training, cancer centers may be able to significantly reduce the ongoing cost of identifying and enrolling patients for smoking cessation treatment.

The observed cross-site heterogeneity in costs and benefits of the IT intervention has implications for stakeholders. Although the overall ICQ estimate for IT vs usual care was $9866, the Site B results (ICQ IT vs usual care: $5408, base case; $3786, best case) show that lower ICQs are possible. Site B used master’s level counselors and built customized workflows de novo*.* In contrast, Site A already had a high-volume smoking cessation program with a preexisting integrated workflow akin to ST in place. They also employed nurse practitioners as counselors (nurse practitioner wages are more than twice those of master’s level counselors) and devoted more time to routine clinical documentation requirements. While it is possible that clinics employing nurse practitioners and other licensed independent practitioners may be able to recoup clinical revenue by billing for cessation services, coverage for smoking cessation is modest, and additional work is needed to overcome administrative burdens associated with seeking insurance coverage and reimbursement.^34^

### Strengths and Limitations

Strengths of the current study are cost data collected contemporaneously and in conjunction with the trial being assessed, cross-site comparisons of results, and the use of probabilistic and deterministic sensitivity analyses. Nevertheless, the present findings should be understood in the context of certain analysis choices and limitations. Personnel costs were based on national median wages; the cost-effectiveness of the intervention will vary depending on locally prevailing health care wages. Similarly, this trial was conducted at NCI-accredited comprehensive cancer centers. Each has a clinical infrastructure, including a well-developed electronic health record, that is taken as given in this evaluation. Other health systems might incur additional costs depending on local infrastructure. Stakeholders considering adoption of the IT intervention should consider how their local clinical context will affect implementation costs, including whether their current cessation program more closely resembles ST or usual care. The present findings emphasize the benefit of seeing the full range of costs and benefits across sites, providing a more complete picture of how each may vary when interventions are implemented in practice by individual organizations.

A cancer center’s perspective was taken to inform the stakeholders most directly in control of the decision and resources needed to offer smoking cessation treatment to individuals being treated for cancer. However, this omits changes in lifetime health care costs and patient costs, such as copays and the time commitment required to obtain smoking cessation treatment. In addition, the analysis was conducted in the context of a clinical trial whose control group was chosen to focus inference on clinical effectiveness rather than cost-effectiveness. Although estimates of the ICQ for IT vs usual care are presented under a range of assumptions, it was not possible to determine the relative likelihood of the alternative scenarios.

## Conclusions

The Smokefree Support Study established the effectiveness of IT relative to ST for patients who smoke entering cancer care; the present findings suggest that the cost-effectiveness of IT relative to both ST and usual care is favorable. Some sites may be able to deliver the IT intervention at a lower ICQ through streamlined identification of smokers (for example, using health information technology) as well as alternate staffing and workflow decisions. Because tobacco use adversely affects cancer treatment outcomes, the IT intervention is likely to be highly cost-effective and potentially cost saving.

## Notes

Supplement. **eTable 1. **Descriptions of Simulation Parameters: Training **eTable 2. **Descriptions of Simulation Parameters: Patient Identification Activities Conducted by Research Assistant **eTable 3. **Descriptions of Simulation Parameters: Counseling Delivery **eTable 4. **Descriptions of Simulation Parameters: Medication Costs (per Fill) **eTable 5. **Descriptions of Simulation Parameters: Other Resources **eTable 6. **Descriptions of Simulation Parameters: Wages **eTable 7. **Descriptions of Simulation Parameters: Stochastic Parameters
