# Supplementary material for: Cost-effectiveness of Implementing Smoking Cessation Interventions for Patients With Cancer
Source: JAMA Netw Open. 2022 Jun 9;5(6):e2216362. doi: 10.1001/jamanetworkopen.2022.16362 (PMC9185176; doi:10.1001/jamanetworkopen.2022.16362)
Supplement: Supplement. — eTable 1. Descriptions of Simulation Parameters: Training eTable 2. Descriptions of Simulation Parameters: Patient Identification Activities Conducted by Research Assistant eTable 3. Descriptions of Simulation Parameters: Counseling Delivery eTable 4. Descriptions of Simulation Parameters: Medication Costs (per Fill) eTable 5. Descriptions of Simulation Parameters: Other Resources eTable 6. Descriptions of Simulation Parameters: Wages eTable 7. Descriptions of Simulation Parameters: Stochastic Parameters [file jamanetwopen-e2216362-s001.pdf]

Supplemental Online Content

Levy DE, Regan S, Perez GK, et al. Cost-effectiveness of implementing smoking cessation interventions for patients with cancer. *JAMA Netw Open.* 2022;5(6):e2216362. doi:10.1001/jamanetworkopen.2022.16362

eTable 1. Descriptions of Simulation Parameters: Training

eTable 2. Descriptions of Simulation Parameters: Patient Identification Activities Conducted by Research Assistant

eTable 3. Descriptions of Simulation Parameters: Counseling Delivery

eTable 4. Descriptions of Simulation Parameters: Medication Costs (per Fill)

eTable 5. Descriptions of Simulation Parameters: Other Resources

eTable 6. Descriptions of Simulation Parameters: Wages

eTable 7. Descriptions of Simulation Parameters: Stochastic Parameters

This supplemental material has been provided by the authors to give readers additional information about their work.

© 2022 Levy DE et al. *JAMA Network Open.*

eTable 1. Descriptions of Simulation Parameters: Training

Tasks	*Quantity/duration*	*Cost*
Initial		
Training courses	45 hours per counselor	$1150 tuition plus counselor time and ancillary (e.g., transportation) costs
One-on-one motivational interviewing training	4 hours per counselor	Counselor (trainee) time, psychologist (trainer) time
Program staff training	40 hours x 1 research assistant	Research Assistant (trainee) time, psychologist (trainer) time
Ongoing		
Counselor supervision	1 hour per counselor per week	Counselor time, psychologist time

Note: Site A employed 3 counselors, Site B employed 2 counselors.

© 2022 Levy DE et al. *JAMA Network Open.*

eTable 2. Descriptions of Simulation Parameters: Patient Identification Activities Conducted by Research Assistant[a]

	Site A		*Site B*	
Pre-enrollment activities	*N*	*Time (hours)*	*N*	*Time (hours)*
Screen daily clinic schedule for potential eligible participants	n/a	n/a	12137	0.02
Chart screen those who are identified as potential eligible participants	3274	0.17	1435	0.13
Introductory phone calls to potential participants (includes multiple calls, missed calls)	7954	0.17	3312	0.08
Recruitment activities at clinic visit	582	0.15	485	0.5

[a] In practice, role might be fulfilled by a social worker and/or medical assistant.

© 2022 Levy DE et al. *JAMA Network Open.*

eTable 3. Descriptions of Simulation Parameters: Counseling Delivery

Counseling sessions (counseling and documentation)	*Overall*		*Site A*		*Site B*	
	% Sessions completed	*Average duration (hours)*	*% Sessions completed*	*Average duration (hours)*	*% Sessions completed*	*Average duration (hours)*
Session 1	100%	1.23	100%	1.87	100%	0.84
Session 2	89%	0.82	86%	1.31	91%	0.54
Session 3	76%	0.77	74%	1.21	77%	0.51
Session 4	65%	0.75	55%	1.26	70%	0.50
Session 5	73%	0.85	67%	1.42	76%	0.53
Session 6	56%	0.78	44%	1.36	63%	0.52
Session 7	54%	0.69	25%	1.52	72%	0.51
Session 8	51%	0.65	18%	1.36	71%	0.54
Session 9	61%	0.76	47%	1.32	69%	0.52
Session 10	51%	0.66	33%	1.15	62%	0.50
Session 11	47%	0.60	25%	1.12	61%	0.46
Call attempts						
Ad hoc counseling/ urgent issues: psychologist time	Occurs for 3.3% of completed sessions	0.50	Occurs for 6.4% of completed sessions	0.50	Occurs for 1.9% of completed sessions	0.50
Ad hoc counseling/ urgent issues: counselor time	Occurs for 1.9% of completed sessions	0.17	Occurs for 0.8% of completed sessions	0.17	Occurs for 2.4% of completed sessions	0.17

© 2022 Levy DE et al. *JAMA Network Open.*

eTable 4. Descriptions of Simulation Parameters: Medication Costs (per Fill)[b]

	Overall, $ (n)	*Site A, $ (n)*	*Site B, $ (n)*
Varenicline, 0.5mg and 1mg starter pack, 53 tablets	$282.39 (19)	$282.39 (7)	$282.39 (12)
Varenicline, 1mg, continuing, 56 tablets	$385.20 (27)	$385.20 (12)	$385.20 (15)
Bupropion, 150mg, 60 tablets	$229.12 (15)	$229.12 (2)	$229.12 (13)
Nicotine patch, 7mg or 14mg or 21mg, 14 patches	$51.96 (156)	$51.96 (43)	$51.96 (113)
Nicotine lozenges, 2mg or 4mg; 81 pieces	$22.19 (163)	$22.19 (36)	$22.19 (127)
Labor[c] and shipping	$27.35 (380)	$17.53 (100)	$47.49 (280)

[b] Prescription medication (varenicline, bupropion) costs are 2018 average wholesale prices minus 20% from the MicroMedex Red Book. Over-the-counter medication (nicotine patch, lozenge) are based on the lowest price identified through online vendors (e.g., CVS, Wallgreens, Target, Walmart). Costs were the same for both sites.
[c] Research assistant time [both sites] and psychiatrist time [Site B medication reconciliation only]

© 2022 Levy DE et al. *JAMA Network Open.*

eTable 5. Descriptions of Simulation Parameters: Other Resources

	Overall	*Site A*	*Site B*
Written patient materials (printing, postage)[d]	$27.26 per enrolled participant	$48.67 per enrolled participant	$13.72 per enrolled participant
Office space and related overhead	$281.25 per FTE per month	$281.25 per FTE per month	$281.25 per FTE per month

[d] Per enrolled participant costs include costs of screening participants who did not enroll; site-specific variation reflects the differences in enrollment yield at the two sites.

© 2022 Levy DE et al. *JAMA Network Open.*

eTable 6. Descriptions of Simulation Parameters: Wages[e]

Wages (32% fringe included)	*Rate*	*Role*	*Notes*
Psychologist	$82.02	Training and supervising the counseling and administrative staff	Wage is 90th percentile of national average, reflecting the training/expertise required for the role.
Social worker	$28.54	Counselors	Site B only
Nurse practitioner	$67.93	Counselors	Site A only
Psychiatrist	$134.73	Medication reconciliation	Site B only; mean but not median wage is reported for psychiatrists – median is imputed based on the ratio mean:median wages for other physicians
Research Assistant	$29.59	Program administration and support, participant identification and recruitment, medication delivery	In practice, role might be fulfilled by a social worker and/or medical assistant.

[e]Based on 2018 U.S. national median wages obtained from the U.S. Bureau of Labor Statistics (www.bls.gov/oes/current/oes-nat.htm), except where noted.

© 2022 Levy DE et al. *JAMA Network Open.*

eTable 7. Descriptions of Simulation Parameters: Stochastic Parameters[f]

Parameter	*Distribution*	*Source*	*Notes*
Number of medications filled	Beta	Assumed	Separate draws for each medication for each simulated analysis
Medication cost			Independent cost multipliers for each medication.
Wage multiplier	Beta	Assumed	One multiplier applies to all wages in a simulated analysis (i.e., variation is correlated across wages)
Written patient materials	Beta	Assumed	
Office space and related overhead	Beta	Assumed	
Time to screen patients	Beta	Assumed	
Number of patients screened	Beta	Assumed	
Counseling prep time	Beta	Assumed	
Counseling sessions attended	Normal	Observed mean and standard error	
Duration of counseling sessions	Normal	Observed mean and standard error	
Duration of documentation time	Normal	Observed mean and standard error	
Call attempts	Beta	Assumed	

[f] Where distributions were assumed we used multipliers drawn from a beta distribution to add stochastic variability to a simulation parameter. All multipliers randomly drawn from a beta distribution with a mean of 1 and a 99% confidence interval of 0.9 to 1.1. As an example, the mean cost of nicotine patches was $51.96; the value used in a simulation was $51.96 multiplied by the randomly-drawn multiplier.

© 2022 Levy DE et al. *JAMA Network Open.*
